# Treating Schizophrenia with DOTS in Developing Countries: Author's Reply

**DOI:** 10.1371/journal.pmed.0040285

**Published:** 2007-09-25

**Authors:** Saeed Farooq

I am grateful to Souza et al. [1] for taking interest in our article [2] and pointing out some very relevant points. I agree with the authors that despite the fact that the World Health Organization and many other agencies have advocated that mental health care in developing countries be integrated into primary care, there has been no real progress. We need to think about the real causes for this failure. Lack of commitment by governments is only a partial explanation. We, the mental health professionals practicing in developing countries, must also accept responsibility. One of the major reasons is that we have not been able to formulate simple interventions that can be implemented at the primary care level as a public health measure. The approach based on DOTS for treating schizophrenia is one such intervention.

Most of the issues raised by Souza et al. are the problems that we are likely to face in applying an approach developed basically for an infectious disease to a chronic noncommunicable disease. I would like to stress that, as mentioned in the article, the approach is based on the principles of DOTS, not on applying DOTS exactly as practiced in tuberculosis control to the treatment of schizophrenia. I agree with the authors that it will need considerable modifications before it can be applied to a chronic disorder like schizophrenia. They have pointed out several issues and I would like to address these.

1. Health workers would definitely need to be better trained under the supervision of mental health professionals to apply this approach in primary care.

2. I very much appreciate the work of the authors and agree that passive case finding is not an option. This will result in the plight of patients mentioned in their letter. One of the benefits of the approach suggested in our article is that as a result of an intervention available at the public health level there will be greater awareness of severe mental illness. Consequently there will be earlier recognition of these cases in the community. As mentioned in the article, this should also result in reduced stigma for the disorder.

3. It should be possible to provide a standard regimen for treatment of schizophrenia based on the essential psychotropic drugs. We were able to develop this for our pilot project and are also using the same approach in our randomized controlled trial [3].

4. One of the major reasons for advocating this approach is that it could ensure free supplies of the drugs as a part of a DOTS program. One of five essential components of the DOTS strategy is government commitment to providing drugs free of cost. This is the cornerstone of the strategy suggested in our article.

5. Monitoring and tracking of patients is important but need not stretch primary care workers beyond capacity, as schizophrenia is a low prevalence disorder. As explained in the article, the implementation of DOTS would be for a two-year period. The community can only be involved if we can offer effective interventions for those suffering from this chronic and disabling disorder.

The approach suggested in our article represents an attempt to bring mental health into the public health arena. Schizophrenia is a low prevalence disorder, for which effective interventions are available and can be implemented at the community level. It therefore represents an ideal disorder for intervention based on DOTS. Applying an approach developed essentially for a disorder which has a time-limited course and is high on the public health agenda to a disorder which is noncommunicable and runs a much protracted course demands a paradigm shift. There are examples of similar approaches in other noncommunicable diseases. Insulin Demonstration projects, which have been initiated to improve access to insulin by the International Diabetes Foundation Task Force, can provide good models [4]. Small scale programs based on the model suggested in our article should be developed locally in developing countries before we can expect governments to support them. Organizations such Médecins Sans Frontières are ideally suited to develop programs like these. One size may not fit all but we can make a suitable size for a great majority.

## References

[pmed-0040285-b001] Souza R, Yasuda S, Cristofani S (2007). Treating schizophrenia with DOTS in developing countries: One size does not fit all. PLoS Med.

[pmed-0040285-b002] Patel V, Farooq S, Thara R (2007). What is the best approach to treating schizophrenia in developing countries?. PLoS Med.

[pmed-0040285-b003] [No authors listed] (2007). Supervised treatment of schizophrenia: A randomized controlled trial. ID NCT00392249. NCT00392249.

[pmed-0040285-b004] International Diabetic Federation (2004). Insulin Demonstration project. http://www.idf.org/e-atlas/home/index.

